# Diversity and Dynamics of a Widespread Bloom of the Toxic Dinoflagellate *Alexandrium fundyense*

**DOI:** 10.1371/journal.pone.0022965

**Published:** 2011-07-29

**Authors:** Deana L. Erdner, Mindy Richlen, Linda A. R. McCauley, Donald M. Anderson

**Affiliations:** 1 University of Texas Marine Science Institute, Port Aransas, Texas, United States of America; 2 Woods Hole Oceanographic Institution, Woods Hole, Massachusetts, United States of America; Mt. Alison University, Canada

## Abstract

Historically, cosmopolitan phytoplankton species were presumed to represent largely unstructured populations. However, the recent development of molecular tools to examine genetic diversity have revealed differences in phytoplankton taxa across geographic scales and provided insight into the physiology and ecology of blooms. Here we describe the genetic analysis of an extensive bloom of the toxic dinoflagellate *Alexandrium fundyense* that occurred in the Gulf of Maine in 2005. This bloom was notable for its intensity and duration, covering hundreds of kilometers and persisting for almost two months. Genotypic analyses based on microsatellite marker data indicate that the open waters of the northeastern U.S. harbor a single regional population of *A. fundyense* comprising two genetically distinct sub-populations. These subpopulations were characteristic of early- and late-bloom samples and were derived from the northern and southern areas of the bloom, respectively. The temporal changes observed during this study provide clear evidence of succession during a continuous bloom and show that selection can act on the timescale of weeks to significantly alter the representation of genotypes within a population. The effects of selection on population composition and turnover would be magnified if sexual reproduction were likewise influenced by environmental conditions. We hypothesize that the combined effects of differential growth and reproduction rates serves to reduce gene flow between the sub-populations, reinforcing population structure while maintaining the diversity of the overall regional population.

## Introduction

Because of the apparent lack of barriers to dispersal and therefore gene flow in the marine pelagic environment, it would be reasonable to hypothesize that phytoplankton species comprise a single, largely unstructured population. This assumption was first challenged by Gallagher (1980) [1], who conducted the first quantitative study of microalgal population genetics, revealing seasonal changes in the composition of populations of *Skeletonema costatum*, a marine diatom. In the intervening thirty years, a variety of studies have used different molecular markers to examine the diversity of microalgal populations over a range of temporal and spatial scales. Population substructure has been detected within a single sample [2] as well as between geographically disjunct populations [3], [4]. Population succession has been observed in blooms of the same species occurring one month apart [5]; yet no differentiation was found in eleven daily samples from a single bloom or samples collected in different years from the same location [6]. The latter study was the only one to sample over the entire course of a phytoplankton bloom. Due to their intensity and episodic nature, bloom events can have a disproportionately large impact on the ecology and biogeochemistry of an ecosystem. Thus, understanding the diversity and dynamics of phytoplankton populations, and of blooms specifically, is key to understanding the overall ecological significance of algal blooms.

The dinoflagellate *Alexandrium fundyense* is an ideal system for studying the diversity and dynamics of algal blooms. It is a cosmopolitan genus [7], and species within the *catenella/fundyense/tamarense* species complex are found in temperate coastal waters worldwide. Because of its ability to produce potent neurotoxins and therefore cause harmful algal blooms, it is subject to intense monitoring with respect to population dynamics and accompanying environmental conditions (e.g. [8]). The physiology, ecology, and toxicity of this organism have also been extensively investigated in both laboratory and field contexts, making it one of the most well-studied marine unicellular algal species. In addition, the life cycle of *Alexandrium fundyense* involves an annual alternation between asexual and sexual reproduction [9]. Binary fission is the primary mode of reproduction during vegetative growth (i.e. during blooms) whereas mating is triggered presumably in response to unfavorable conditions (i.e. at the end of blooms), with vegetative cells not overwintering in the water column. Thus, population genetic studies of *Alexandrium* blooms have the potential to help us understand not only how algal blooms form and persist, but also how a haplo-diploid life cycle impacts the diversity of algal populations.

Here we describe the temporal and spatial changes in the genetic composition of a continuous bloom of *Alexandrium fundyense* in the Gulf of Maine, USA. This bloom occurred from May to July of 2005 and, at its greatest extent, affected over 700 km of coastline [10]. Microsatellite markers were used to genotype 171 clonal isolates established from samples collected at six locations spanning the spatial and temporal extent of the bloom. The purpose of the study was to address several questions regarding the formation of *A. fundyense* blooms in a relatively large, open marine basin with extensive circulation. First, are *A. fundyense* blooms, which can extend for hundreds of kilometers and last for weeks to months, composed of a single unstructured population or multiple, genetically distinct (sub) populations? Second, is the diversity and composition of the bloom static, or does it change over time? If so, on what scale? Lastly, what can the diversity and dynamics of this bloom tell us about how *A. fundyense* populations are maintained year-to-year, given their annual alternation in life cycle? Our results show that the beginning of the 2005 *A. fundyense* bloom was characterized by the presence of a common and diverse sub-population at three geographically separated sampling sites. Samples collected later in the bloom were genetically differentiated from these early samples, with the change in population composition occurring on the timescale of approximately four weeks. The temporal changes observed during this study provide clear evidence of succession during a single bloom and show that selection can act on the timescale of weeks to significantly alter the representation of genotypes within a population. If environmental conditions drive both the changes in population composition and the induction of sexual reproduction, the resulting interactions between succession and reproduction will reinforce the genetic differentiation between sub-populations while maintaining the overall diversity of the regional population.

## Materials and Methods

### Ethics Statement

No permits are required for collection of water samples in U.S. coastal waters.

### Cultured strains

**Figure 1:**
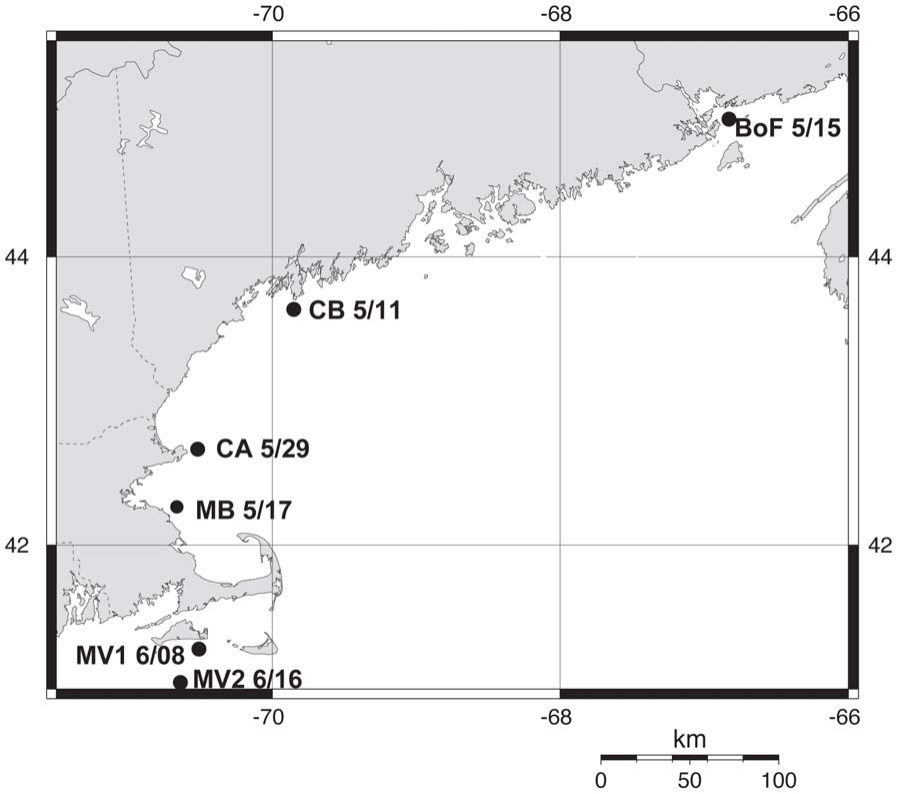
Sample collection dates and locations. Map of the northeastern U.S. including the Gulf of Maine, showing the collection locations and dates of samples analyzed in this study.

A total of 171 clonal strains were used in this study. Strains were isolated from six different locations throughout the Gulf of Maine; isolation dates and locations are shown in Figure 1. Samples from Casco Bay (CB), Bay of Fundy (BoF), and Massachusetts Bay (MB) were collected during cruise OC412 (R/V Oceanus); the sample from Cape Ann (CA) was collected on cruise Ti_096 (R/V Tioga); the first sample from Martha's Vineyard (MV1) was collected on cruise Ti_099; and the second sample from Martha's Vineyard was collected on cruise Ti_103. All strains were established by manual isolation and transfer of a single *A. fundyense* cell to 200 µL of f/2-Si medium [11] in each well of a 48-well plate. After isolates reached ≥20 cells per well, they were transferred to 25 mL of fresh medium in a glass culture tube. The average success rate of the isolations was 38±8.3% (range 28–50%), defined as the percent of cells isolated that survived sufficiently long in clonal culture to be genotyped. There are two morphologically similar dinoflagellate species in the Gulf of Maine, and occasionally cells of these species would be picked. These cells were not propagated into larger cultures, nor were they considered in the success rate calculations. This happened infrequently, but in plates where this occurred the success rates would be higher by 1–2% than the rates given above.

### DNA Extraction

For DNA extraction, strains were grown in medium consisting of 0.45 µm-filtered and autoclaved natural seawater (Vineyard Sound, MA, salinity 31 psu) enriched with modified f/2-Si nutrients. Cultures were grown at 15°C on a 14∶10 light∶dark cycle with cool-white fluorescent illumination of approximately 100 µmol photons•m^−2^•s^−1^. When cultures reached mid-exponential phase, approximately 20 mL was harvested by centrifugation (3000× g for 5 min). DNA was extracted from cell pellets using the Qiagen DNeasy Tissue Kit (Valencia, CA, USA). The protocol was modified by the addition of a mechanical breakage step, in which a double volume of lysis buffer and 0.5 mm silica-zirconium beads (BioSpec Products, Inc. Bartlesville, OK, USA) were added to the cell pellets followed by vortex mixing for 1 minute at maximum speed. All subsequent buffer volumes were also doubled, prior to application of the lysate to the Qiagen column. Wash buffer volumes were not increased, and DNA was eluted in 2×100 µL of elution buffer. Whole genomic DNA was stored at −20°C until used for PCR amplification.

### Microsatellite genotyping

Of the microsatellite loci isolated by Nagai et al. (2004) [12], eleven of thirteen loci were tested with a set of culture collection strains of *A. fundyense*, to determine the most suitable markers (data not shown). Five loci were chosen based upon the number of alleles observed and the percent of amplifying samples, and these five primer sets were used to determine the genotype of all of the strains used in this study. PCR amplification reactions contained 5 ng of template DNA, 0.5 mM of each dNTP, 0.25 µM of each designed primer pair, with one primer labelled with D4-WellRED (Sigma-Proligo, St. Louis, MO, USA), 1× PCR buffer (10 mM Tris-HCl, pH 8.3, 500 mM KCl, 15 mM MgCl_2_, 0.01% w/v gelatin), and 0.25 U of Ampli *Taq* Gold (Applied Biosytems Inc.) to a total volume of 10 µL. The PCR cycling conditions were as follows: 5 min at 96°C, 45 cycles of 45 s at 95°C, 45 s at 54°C (Atama15, 16, 27, 29) or 58°C (Atama39), and 1 min at 72°C, and a final elongation for 1 min at 72°C. PCR products were diluted with nuclease-free water and 1 µL of diluted product was mixed with 500 LIZ Size Standard and Hi-Di Formamide, and then analyzed using an ABI 3730xl DNA Analyzer. Allele sizes were determined using the program FPMiner (2005, BioinforSoft LLC, Beaverton, OR, USA).

### Microsatellite Data Analysis

We performed a variety of tests to characterize the diversity and potential structure present in the bloom assemblage. The diversity was characterized by computing the molecular diversity and proportion of unique haplotypes within the samples. The presence of linked loci, a common consequence of predominantly clonal reproduction, was also assessed. A primary question in this study concerned the potential presence of genetic structure within the bloom. Genetic distances between samples were quantified by calculating the pairwise Fixation Index (F*_ST_*) and genetic distance between samples based on allele frequencies, and further tested using an exact G test. Lastly, the membership of individuals and samples in the different sub-populations was determined using Bayesian assignment tests, and their similarity was visualized by Principal Components Analysis.

Assessment of the number of unique haplotypes and those shared between samples was done using Arlequin v 3.5.1.2 [13]. Calculation of standard molecular diversity indices, e.g., number of alleles and haplotype diversity, was performed in PopGene v 1.31 [14]. The presence of multilocus linkage disequilibrium (I_A_^S^, a measure of linkage) was investigated using the Monte Carlo method in LIAN v 3.5 (http://adenine.biz.fh-weihenstephan.de/cgi-bin/lian/lian.cgi.pl; [15]) with 10,000 resamplings, whereas linkage disequilibrium between pairs of loci was determined in Arlequin v 3.5.1.2. The partitioning of genetic variance within and among samples was determined using analysis of molecular variance (AMOVA) - over all loci and per locus – with distance based on the number of different alleles, implemented in Arlequin v3.5.1.2. Comparisons of the genetic differentiation between samples were made in several ways. Populations pairwise *F*_ST_ values were calculated in Arlequin using 10,000 permutations to test significance. An exact G test (Fisher's test) for genic differentiation (null hypothesis = no differentiation) was performed using GenePop v4.0.10 (http://genepop.curtin.edu.au/; [16]), with Markov Chain parameters of 1000 dememorisation steps and 100 batches with 1000 iterations per batch [17]. To visualize the relationships between samples, a dendrogram based on Nei's (1972) genetic distance was constructed using the UPGMA algorithm implemented in TFPGA v1.3 [18]. PCAGEN [19] was used to perform a principal components analysis of *F*_ST_ values, with 10000 randomisations used to determine the significance of the inertia of each axis. For all instances of multiple tests, significance levels were adjusted using a sequential Bonferroni procedure. The presence of genetic structure and admixture in our samples was assessed using STRUCTURE 2.3.2 [20]. Simulations were performed using the admixture model with and without the locprior option, assuming correlated allele frequencies among populations. Ten runs were performed at each K value from 1 to 10, with a burn-in period of 10,000 steps and 10,000 Markov Chain Monte Carlo repetitions. The log likelihoods of the data from the 20 runs at a given K were averaged, and the resulting values were used to calculate the statistic ΔK [21]. When plotted against K, the statistic ΔK is expected to show a mode at the most likely value of K.

### Environmental and Drifter Data

Temperature, salinity, and nutrient conditions at our sampling sites are listed in Table 1. Data for samples CB, BoF, MB, CA, and MV2 were obtained from http://science.whoi.edu/users/olga/alex_surveys_2005/Alexandrium_Surveys_2005.html. For sample MV1, no nutrient data were available, but temperature and salinity were obtained from the R/V Tioga data archive, http://www.whoi.edu/page.do?pid=8439. Drifter data was obtained from the Gulf of Maine Drifter Track archive (http://gisweb.wh.whoi.edu/cgi-bin/ioos/index_drift_dods.pl) maintained by Jim Manning at the NOAA Northeast Fisheries Science Center Oceanography Branch [22]. Water transit times between BoF and CB were determined from drifter #55465, from CB to CA from drifter #55382, and from CA to MV from drifter #55202.

**Table 1:** Sample dates, geographic coordinates and environmental data.

Sample	Date	Latitude (°N)	Longitude (°W)	Temp (°C)	Salinity (psu)	NH_4_ (µM)	Si (µM)	PO_4_ (µM)	NO_2_+NO_3_ (µM)
**CB**	05/11/05	43.6992	69.8952	7.9	27.48	12.3	10.5	0.2	2.2
**BoF**	05/15/05	44.9828	66.8250	5.1	29.89	0.5	11.2	0.4	8.5
**MB**	05/17/05	42.2802	70.7843	9.2	29.70	2.0	0.85	0.2	0.1
**CA**	05/29/05	42.6942	70.4852	8.8	29.77	2.1	3.55	0.4	1.7
**MV1**	06/08/05	41.3260	70.5192	14.2	30.31	ND	ND	ND	ND
**MV2**	06/16/05	40.9905	70.5687	8.9	31.32	0.7	4	0.3	0.6

## Results

### Genotypic Data

The microsatellite markers used in this study were developed using *A. tamarense* strains of the NorthAmerican/Group I ribotype from Japan [12]. *Alexandrium fundyense* in the northeastern U.S. belong to this same clade, and so it was expected that the microsatellite markers would be generally applicable to strains from the North American/Group I ribotype [4]. Of the 13 microsatellite loci isolated by Nagai et al. (2004) [12], only 5 loci showed >90% amplification success with a set of test strains, despite multiple PCR attempts (data not shown). Single bands were observed at each locus for all individuals, consistent with the haploid nature of vegetatively growing *A. fundyense* cells. All five loci were polymorphic in all samples, except for locus Atama27 in sample MV1 (Table S1). The number of alleles per locus ranged from 3 to 12, and the genetic diversity per locus varied from 0.19 to 0.84, with an average over all loci of 0.54±0.13. Locus Atama16 had the highest diversity (0.83, 11 alleles), followed by Atama23 (0.72, 9 alleles), and Atama15 (0.69, 12 alleles). Loci Atama27 and Atama39 had a diversity of 0.19 and 0.24 (4 and 3 alleles), respectively. Neither single- nor multilocus linkage disequilibrium was detected (I_A_^S^ = 0.0024, p = 0.394). The overall amplification success was 95% of individuals, with nine strains having missing data at a single locus each. Of the 171 strains analyzed, 119 (70%) had unique five-locus genotypes. The proportion of unique multilocus genotypes per sample ranged from 83 to 92% (Supplementary Table S1), and all samples shared at least one genotype with every other sample. None of the genotypes were common to all samples.

### Genetic Differentiation

Results of the overall AMOVA analysis showed that most of the genetic variation (95%) was found within samples (Table 2). When the five loci were examined separately, only locus Atama15 showed greater variation among samples as compared to the aggregate (16% vs. 5%); at the other four loci virtually all of the variance (≥99%) occurred within samples (Table 3). Of the twelve alleles detected at locus Atama15, one allele (size 242, 23% of all alleles) was found only in samples CA, MV1 and MV2 while another (size 241, 9.4% of all alleles) was found only in samples MB, BoF, CB, and CA.

**Table 2:** Analysis of molecular variance within and among samples from the Gulf of Maine.

Source of variation	d.f.	Sum of squares	Variance	% of variation
**Among samples**	5	15.001	0.06158 V_a_	4.63
**Within samples**	165	209.414	1.26918 V_b_	95.37
**Total**	170	224.415	1.33075	

d.f. = degrees of freedom.

**Table 3:** Per locus AMOVA within and among samples from the Gulf of Maine.

	Among samples				Within samples			
Locus	SSD	d.f.	V_a_	%var	SSD	d.f.	V_b_	%var
**Atama15**	9.43	5	0.056	15.9	49.22	165	0.298	84.1
**Atama16**	2.43	5	0.003	0.60	68.52	165	0.415	99.4
**Atama23**	2.40	5	0.004	1.21	58.63	164	0.358	98.8
**Atama27**	0.51	5	0.000	0.51	14.48	162	0.089	99.5
**Atama39**	0.28	5	−0.002	−1.96	19.36	163	0.119	102.0

SSD = sum of squares.

V = variance.

%var = percent of total variance.

**Figure 2:**
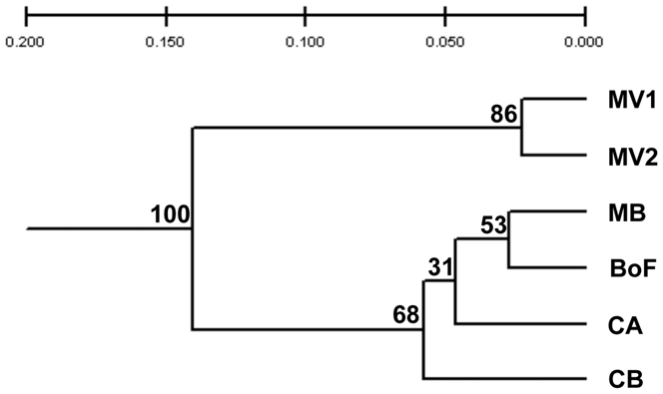
UPGMA dendrogram based on Nei's (1972) genetic distance between the six samples. One thousand bootstrap permutations were conducted; percent of similar replicates is shown above the nodes.

**Figure 3:**
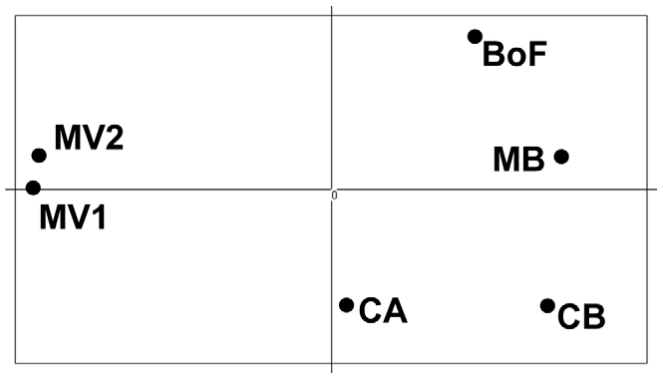
Principal Component Analysis of allele frequencies of the six bloom samples. Only the first (horizontal) axis is significant: percent inertia = 64%, F_ST_ = 0.017, p = 0.009. Axis 2: percent inertia = 16%, F_ST_ = 0.004, p = 0.971.

**Figure 4:**
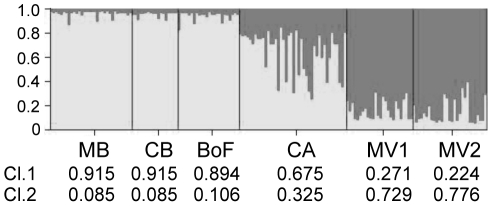
Population structure determined by Bayesian cluster analysis. Results of STRUCTURE analysis are presented for K = 2. The bar plot shows the proportion of membership in Cluster 1 (light gray) or Cluster 2 (dark gray) for each individual. Samples are delineated by vertical black lines, and the average proportion of membership in each cluster by sample is listed underneath the sample names.

Relationships among samples were examined in several ways. Two types of pairwise comparisons – *F*_ST_ and Fisher's exact test - were performed to determine the presence of genetic differentiation between samples, with somewhat different results (Table 4). In both tests, samples MV1 and MV2 were significantly different from samples MB, CB, and BoF. Sample CA was not significantly different from MB, CB, and BoF in either test, and the Fisher's test did not reject the hypothesis of panmixia between CA and both MV samples. In contrast, the *F*_ST_ value between CA and MV2 (but not MV1) was significant. The genetic similarity between the samples was assessed using Nei's (1972) genetic distance to construct a dendrogram of the six samples (Figure 2). The dendrogram identifies two clusters of samples: a highly supported group containing the two MV samples and a second well-supported group comprising MB, BoF, CA, and CB. We further examined the relationships between the samples via principal component analysis of their allele frequencies (Figure 3). Similar to the UPGMA analysis, the PCA separates the samples into two general groups: MV1+MV2, and MB+BoF+CB+CA, although sample CA occupies an intermediate position between the two groups. The relationship of sample CA to early- and late-bloom samples was also examined using Bayesian clustering, to estimate the number of population groups present in our overall sample set (Figure 4). Plots of the ΔK statistic [21] as a function of K showed maximum values at K = 2 and K = 4 (Figure S1).

**Table 4:** Pairwise comparisons of genotypic differentiation between samples.

	MB	CB	BoF	CA	MV1	MV2
**MB**		0.391	0.604	0.125	**Highly sig.**	**Highly sig.**
**CB**	0.005		0.247	0.170	**Highly sig.**	**0.0008**
**BoF**	−0.011	0.013		0.059	**Highly sig.**	**Highly sig.**
**CA**	0.009	0.020	0.020		0.008	0.007
**MV1**	**0.096**	**0.120**	**0.083**	0.043		0.512
**MV2**	**0.097**	**0.106**	**0.084**	**0.048**	−0.011	

Lower diagonal: F*_ST_* values. Upper diagonal: p-values of the Fisher's exact test. Bold type denotes test results significant at P<0.05 after Bonferroni correction for 15 multiple tests.

## Discussion

### Genetic diversity of the bloom

The number of loci used to genotype strains in this study was constrained by our ability to successfully amplify the published *A. tamarense* markers from *A. fundyense*. Per sample, we retrieved 83–92% unique genotypes, and no genotype was observed more than twice in each sample. Overall, our recovery of unique multilocus genotypes (70%) is lower than other studies of *Alexandrium* that have used more loci [2], [4], thus more loci would undoubtedly provide greater resolving power. Nonetheless, we were still able to detect significant differentiation between samples collected from a continuous bloom. We therefore consider our results to be conservative estimates of the diversity and differentiation present in this bloom; the true values are most likely larger than those measured here.

### Bloom chronology

Samples were collected over a 37-day period during an extensive bloom of *A. fundyense* that occurred along the coast of New England, USA (for a detailed description of the bloom see [10] and [23]). As with many toxic blooms, this one was unexpected, so our samples were truly “samples of opportunity” – dictated by the availability of ships for offshore sample collection. The first three samples (CB, BoF, and MB) were collected during a single cruise over the course of one week, and we consider them to be “early-bloom” samples, as the first evidence of shellfish toxicity was detected only one week before. These three locations were widely separated (170–430 km), and each had elevated cell concentrations (>400 cells L^−1^). The next sample, CA, was collected 12 days later and is considered to represent the “mid-bloom”, both geographically and temporally. At that time, cell concentrations were very high (thousands per liter) in that region. The last two samples, MV1 and MV2, were both collected south of the island of Martha's Vineyard, which was the southernmost part of the bloom. Cell concentrations were generally high in this region during June (often >1000 cells L^−1^), even though they were decreasing in areas to the north. These samples were collected eight days (MV1) and ten days (MV2) after the previous samples, and in the weeks following the MV2 sample, cell concentrations across the region declined rapidly until the bloom was considered to be over about four weeks later. Thus, samples MV1 and MV2 are considered to be representative of “late-bloom” conditions, geographically and temporally.

**Figure 5:**
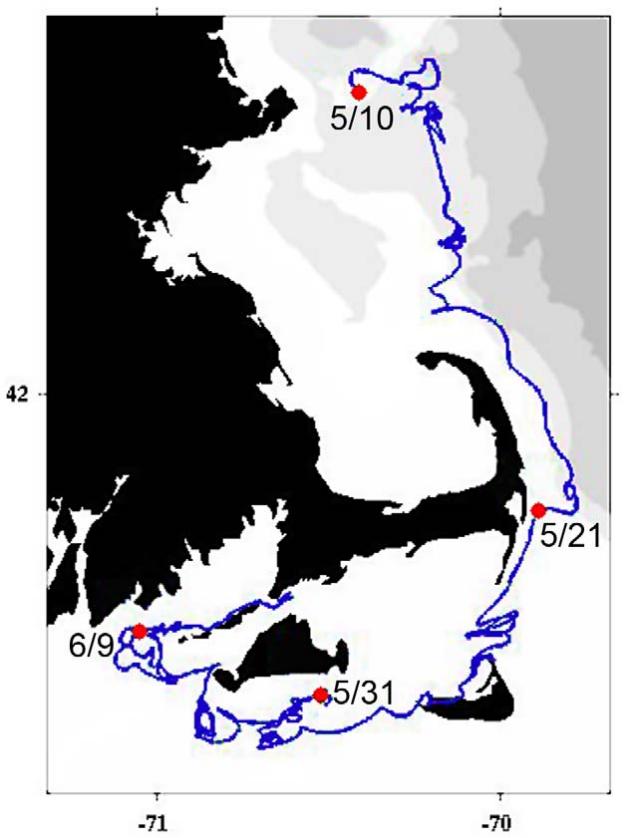
Drifter track showing transit between sampling locations CA and MV. Drifter #55202 was released on 5/10/2005 near Cape Ann, Massachusetts. Drifter location on particular dates is indicated by large circles on the drifter track, labeled with the date the drifter occupied that location.

It is important to keep in mind that while we consider the 2005 event to be a single bloom, we are working in an open basin where water is constantly moving, transporting and potentially mixing cells from different regions. The general circulation pattern in the Gulf of Maine is from north to south along the coast (e.g. see Figure 5), with an along-coast distance from our BoF site to the MV sites of more than 650 km. A number of drifters were deployed in the Gulf of Maine in 2005, and their tracks provide an estimate of the water transit times between sites. It took two weeks for a drifter deployed at BoF on the same day we sampled (15 May) to reach the approximate location of sample CB (29 May). From CB to CA the drifter-derived transit time was on the order of one week (21 May to 29 May), and from CA to MV it was almost three weeks (10 May to 31 May; Figure 5). Thus, any one sample may have cells that originated locally, as well as cells that were transported by currents to that location. In general, the time interval between our sample collections was shorter than the water transit time between the two locations. Thus, while we sampled along the transport pathway of a single bloom, it is unlikely that we sampled the same water mass as it transited the Gulf of Maine. The one exception is sample MV2; it was collected eighteen days after sample CA, and the water transit time between the two locations is estimated at twenty days.

### Population structure

The results of our analyses group the samples into two clusters, the first comprising early- and mid-bloom samples (CB, BoF, MB, and CA) and the second containing only the late-bloom samples (MV1 and MV2). Significant *F*_ST_ values were found in all pairwise comparisons of early- and late-bloom samples (Table 4), ranging from 0.083 (BoF-MV1) to 0.120 (CB-MV1). There are few *F*_ST_ data from *Alexandrium* species with which to compare the extent of the differentiation between our samples. Masseret et al. (2009) [24] calculated significant values of 0.150 to 0.581 for *Alexandrium catenella* populations from France and Japan, and Alpermann et al. (2009) [2] report one significant *F*_ST_ of 0.06 between *Alexandrium tamarense* subgroups (delineated using AFLP) of a single net tow sample. By comparison, the early- and late-bloom samples show greater differentiation than subgroups of a single sample, but less than samples derived from different continents. Because of their hydrographic connection and the level of gene flow observed, we do not consider the early- and late-bloom clusters to represent separate populations, but rather different sub-populations within the regional Gulf of Maine *A. fundyense* population. This designation is supported by the results of the AMOVA analysis (Table 2), which show that between-sample variation accounts for only 5% of the genetic diversity in the region. AMOVA at each locus indicates that Atama15 is driving the genetic differentiation between the different sub-populations (Table 3); at this locus 16% of the variation is found between samples as compared to only 5% overall.

While the early- and late-bloom samples are clearly differentiated, the relationship of the mid-bloom CA sample to late-bloom samples from MV is somewhat unresolved. Neither the Fisher's exact test nor *F*_ST_ distinguishes samples CA and MV1, but the *F*_ST_ between CA and MV2 is significant, whereas the Fishers' test does not reject panmixia between the two samples. At locus Atama15, sample CA shares one unique allele with the early bloom samples (MB, BoF, CB) and another with the late-bloom samples (MV1 and MV2). The intermediate position of sample CA is exemplified by its location in the PCA analysis (Figure 4) and its composition as determined by Bayesian clustering (Figure 2). In the latter analysis, sample CA appears to be admixed to some extent – it contains individuals with allele proportions similar to individuals within both the early- and late-bloom samples – but most individuals in the sample show allele proportions different from the two groups. The mid-bloom sample may therefore represent a transitional stage in the bloom, as the genetic composition is moving from one dominated by the early-bloom sub-population to one dominated by late-bloom genotypes.

The early- and late-bloom sub-populations observed in 2005 may be representative of “northern” and “southern” populations identified in previous studies of *A. fundyense* in the region. A general north-to-south- trend of decreasing toxicity spanning nearly two orders of magnitude has been documented in the GOM, with isolates from the east and north (e.g., the Bay of Fundy) having higher toxin content and different toxin profiles than isolates from the south, including Cape Cod [25], [26]. This toxicity data, combined with differences in bioluminescence and morphological characteristics, led Anderson et al. (1994) [25] to identify two distinct clusters of strains – northern and southern. The microsatellite data support the existence of at least two genetically distinct subpopulations in the region, which requires some kind of separation of the two groups that would reduce gene flow and result in the differentiation of allele frequencies. For example, sub-populations could be separated by geography, the timing of emergence from cysts, growth, or behavior.

Differences in the growth response to environmental conditions appear to play a role in structuring marine diatom populations [27], and one possibility is that differential growth plays a similar role in *A. fundyense* population dynamics. In this scenario, the regional population would comprise a large number of different clonal lineages, which have different growth responses to environmental conditions (e.g. salinity, temperature). All of these lineages are present in each sample, but their proportional representation would be determined by environmental conditions. Because we sampled only 18–42 individuals from each location, we are analyzing only the most abundant lineages in the sample. The genetic differentiation observed would result from selection of different lineages (ecotypes) that were most successful under the conditions at a particular site. Given the general north-to-south coastal circulation pattern in the Gulf of Maine, this scenario requires only a genetically heterogeneous source of cells in the northern part of the region.

Alternatively, there could be geographically separate populations, as suggested by Anderson et al. (1994) [25]. In this scenario, genetically distinct sources of cells would have to exist, and be maintained, both to the north and south of Cape Cod. The northern source would remain relatively isolated, because of circulation patterns. The southern source, however, would be subject to regular inputs of cells from the northern population. These northern genotypes would have to be greatly reduced or removed to maintain the genetic structure; this could be achieved by strong environmental selection against northern strains or by mating incompatibility (e.g., by mismatch in mating triggers or times). This scenario is less parsimonious in that it requires two separating mechanisms, a combination of geography and selection against northern genotypes.

While the clear differentiation between early- and late-bloom samples could be explained by either selection (differential growth) or geography, the “transitional” nature of sample CA argues for selection as the primary factor driving the differentiation of the two sub-populations. Our results show that CA is not significantly differentiated from the early-bloom samples or MV1, yet it shares some affiliation with MV2. Given that CA is located upstream of MV2 in the transport pathway, the presence of both early- and late-bloom genotypes in sample CA could not result from the mixing of water masses; they must exist in sample CA before it is transported south. The change in the proportions of early- and late-bloom genotypes is presumably driven by selection, although the selective factor is not evident in the limited environmental dataset that we have (Table 1). Thus, while environmental conditions seem to be the most likely structuring force, definitively distinguishing between the two hypotheses – geographic populations vs. selection – requires knowledge of the sources of cells (cyst seedbeds, see below) and their genetic composition. At present, there is no data on cell sources south of Cape Cod, although the cyst beds in the Gulf of Maine and Bay of Fundy are well-characterized [28]. Analysis of the genetic composition of the Gulf of Maine cyst sources would demonstrate whether only early-bloom genotypes were present (geographic populations) or both early- and late-bloom genotypes were represented (selection) in the seedbeds.

### Population dynamics

The changes in genetic composition that we observed during this study show that phytoplankton blooms are not static; they comprise a tremendous diversity of strains whose representation shifts over time, presumably in response to changing environmental conditions. Our results contrast somewhat with the findings of Rynearson and Armbrust (2005) [6], who tracked an entire bloom of the diatom *Ditylum brightwellii* and found no significant differentiation between any of their daily samples. An important difference between these two studies is the temporal scale of the bloom. The *Ditylum* bloom lasted only 11 days whereas the 2005 *Alexandrium* bloom persisted for almost 8 weeks, and was tracked for 39 days. While water movement in the Gulf of Maine makes it generally difficult to track a single water mass, or “piece” of the bloom, we do have two samples, CA and MV2, where the sampling interval was roughly equal to the water transit time between the sites. This provides an estimate of the timescale on which genetic differentiation can occur in a planktonic bloom. In the 18-day interval between samples CA and MV2, the samples became sufficiently differentiated that their pairwise *F*_ST_ (although not the result of the Fisher's exact test) was significant. The lack of differentiation observed from *F*_ST_ and Fisher's exact test comparisons of CA and MV1 (10 day sampling interval), and MV1 and MV2 (8 day sampling interval) suggest that the transition from the early-bloom to late-bloom genotypes was in progress, but not yet complete, in samples CA and MV1. The rate of population differentiation should be affected by the relative growth rates of different clonal lineages, i.e. how quickly one lineage can outgrow another, and also the range and rate of change of environmental conditions during a bloom. In the comparison of the *Ditylum* and *A. fundyense* blooms, turnover should be faster in *Ditylum* because of their greater maximum growth rates. However, the *A. fundyense* bloom likely experienced a wider range of environmental conditions, as it transited roughly four degrees of latitude in the course of six weeks, whereas the *Ditylum* bloom occurred in a single coastal embayment. These two factors may balance each other to result in the finding of a common lack of genetic differentiation over ∼10 days in both a diatom and dinoflagellate bloom; however, more studies are needed to determine if this represents a minimum bound for population turnover in marine planktonic algae.

### Life cycle alternation and the maintenance of diversity

The life cycle of *A. fundyense* is characterized by an annual alternation between asexual haploid cells that reproduce by binary fission in the water column, and a resting stage (cyst) that results from the fusion of two haploid gametes. Sexual reproduction requires different mating types and, in the laboratory, is triggered by adverse environmental conditions [29], [30]. Thus, *A. fundyense* cells do not overwinter in the water column, but rather in the sediments as cysts. These resting cysts can remain viable in the sediments for years, and after a mandatory dormancy period, they germinate in response to favorable conditions in the early spring. Because sexual reproduction, and therefore recombination, happens only once a year, changes in the composition of planktonic populations have the potential to affect the diversity of the resting cyst reservoirs that are the source populations of later blooms.

There is little data on the triggers, timing, or magnitude of encystment in natural populations of *A. fundyense*. If it is triggered by unfavorable environmental conditions, as it is in the laboratory, then it has implications for the genetic composition of both the planktonic and resting cyst populations. In the plankton, environmentally-induced encystment could serve to magnify the effects of selection on the proportions of different genotypes by reducing or removing lineages that are less successful under the prevailing conditions. As a result, encystment would occur throughout the bloom, not just at the end, thereby contributing to the diversity of the cyst seedbeds via the asynchronous input of cysts derived from different sub-populations. Induction of sexual reproduction by unfavorable conditions might also serve to reinforce the genetic differences between sub-populations. For example, if the early-bloom sub-population thrives at lower salinities than the late-bloom sub-population, then as a mixed population encounters a region of higher salinity, early-bloom genotypes will be more likely to participate in mating and encystment, while late-bloom genotypes will be favored and remain in the plankton to continue vegetative growth. This would increase the likelihood of genetically similar cells reproducing, effectively reducing gene flow between sub-populations. Thus, sexual reproduction in response to adverse environmental conditions may serve to both reinforce the genetic differentiation between sub-populations while maintaining the overall diversity of the regional population.

### Conclusions

We examined the genetic composition of an extensive bloom of the toxic dinoflagellate *Alexandrium fundyense*, covering hundreds of kilometers and persisting for almost two months. Practical considerations limited the temporal and geographic density of our samples as well as the number of loci analyzed, thus we consider our results to be conservative estimates of the diversity and differentiation present in this bloom. Genotypic analyses based on microsatellite marker data indicate that the open waters of the northeastern U.S. harbor a single regional population of *A. fundyense* comprising at least two genetically distinct sub-populations. These subpopulations were characteristic of early- and late-bloom samples and were derived from the northern and southern areas of the bloom, respectively. We hypothesize that the observed populations structure results from differential environmental selection on the two sub-populations. The effects of selection on population composition would be magnified if sexual reproduction were likewise differentially influenced by environmental conditions. The combined effects of differential growth and encystment would serve to reduce gene flow between the sub-populations, reinforcing population structure while maintaining the diversity of the overall regional population.

## Supporting Information

Figure S1 **Population structure determined by Bayesian cluster analysis for K = 4.** Plots of the statistic ΔK (Evanno et al. 2005) vs. K showed two modes, at K = 2 and K = 4. Results for K = 2 are included in the text, as the presence of two sub-populations is supported by the results of other analyses, whereas the possibility of four sub-populations is inconsistent with the other data. The bar plot shows the proportion of membership in Cluster 1 (red), Cluster 2 (green), Cluster 3 (blue) or Cluster 4 (yellow) for each individual. Samples are delineated by vertical black lines, and the average proportion of membership in each cluster by sample is listed underneath the sample names.
(TIF)

Table S1 **Summary of molecular diversity indices for the six samples analyzed, both per locus and across all loci.** The number of unique haplotypes per sample is listed beside the sample name.
(DOC)

## References

[1] Gallagher JC (1980). Population genetics of *Skeletonema costatum* (Bacillariophyceae) in Narragansett Bay.. Journal of Phycology.

[2] Alpermann TJ, Beszteri B, John U, Tillmann U, Cembella AD (2009). Implications of life-history transitions on the population genetic structure of the toxigenic marine dinoflagellate *Alexandrium tamarense*.. Molecular Ecology.

[3] Evans KM, Bates SS, Medlin LK, Hayes PK (2004). Microsatellite marker development and genetic variation in the toxic marine diatom *Pseudo-nitzschia multiseries* (Bacillariophyceae).. Journal of Phycology.

[4] Nagai S, Lian C, Yamaguchi S, Hamaguchi M, Matsuyama Y (2007). Microsatellite markers reveal population genetic structure of the toxic dinoflagellate *Alexandrium tamarense* (Dinophyceae) in Japanese coastal waters.. Journal of Phycology.

[5] Rynearson TA, Newton JA, Armbrust EV (2006). Spring bloom development, genetic variation, and population succession in the planktonic diatom *Ditylum brightwellii*.. Limnology and Oceanography.

[6] Rynearson TA, Armbrust EV (2005). Maintenance of clonal diversity during a spring bloom of the centric diatom *Ditylum brightwellii*.. Molecular Ecology.

[7] Balech E (1995). The genus *Alexandrium* Halim (dinoflagellata).

[8] Anderson DM, Townsend DW, McGillicuddy DJ, Turner JT (2005). The Ecology and Oceanography of Toxic *Alexandrium fundyense* Blooms in the Gulf of Maine.. Deep-Sea Research Part II: Topical Studies in Oceanography.

[9] Anderson DM, Anderson DM, Cembella AD, Hallegraeff GM (1998). Physiology and bloom dynamics of toxic *Alexandrium* species, with emphasis on life cycle transitions.. Physiological Ecology of Harmful Algal Blooms.

[10] Anderson DM, Keafer BA, McGillicuddy JDJ, Mickelson MJ, Keay KE (2005). Initial observations of the 2005 *Alexandrium fundyense* bloom in southern New England: General patterns and mechanisms.. Deep Sea Research Part II: Topical Studies in Oceanography.

[11] Guillard RRL, Ryther JH (1962). Studies of marine plankton diatoms I. *Cyclotella nana* Hustedt and *Detonula confervacea* (Cleve) Gran.. Canadian Journal of Microbiology.

[12] Nagai S, Lian C, Hamaguchi M, Matsuyama Y, Itakura S (2004). Development of microsatellite markers in the toxic dinoflagellate *Alexandrium tamarense* (Dinophyceae).. Molecular Ecology Notes.

[13] Excoffier L, Laval G, Schneider S (2005). Arlequin ver. 3.0: An integrated software package for population genetics data analysis.. Evolutionary Bioinformatics Online.

[14] Yeh FC, Boyle TJB (1997). Population genetic analysis of co-dominant and dominant markers and quantitative traits.. Belgian Journal of Botany.

[15] Haubold B, Hudson RR (2000). LIAN 3.0: detecting linkage disequilibrium in multilocus data.. Bioinformatics.

[16] Rousset F (2008). GenePop '07: A complete reimplementation of the GenePop software for Windows and Linux.. Molecular Ecology Resources.

[17] Raymond M, Rousset F (1995). An Exact Test for Population Differentiation.. Evolution.

[18] Miller MP (1997). Tools For Population Genetic Data Analysis..

[19] Goudet J (1999). PCAGEN ver. 1.2..

[20] Pritchard JK, Stephens M, Donnelly P (2000). Inference of population structure using multilocus genotype data.. Genetics.

[21] Evanno G, Regnaut S, Goudet J (2005). Detecting the number of clusters of individuals using the software STRUCTURE: a simulation study.. Molecular Ecology.

[22] Manning JP, McGillicuddy D, Pettigrew N, Churchill JH, Incze L (2009). Drifter observations of Gulf of Maine coastal current.. Continental Shelf Research.

[23] He R, McGillicuddy DJ, Anderson DM, Keafer BA (2008). Historic 2005 toxic bloom of *Alexandrium fundyense* in the western Gulf of Maine: 2. Coupled biophysical numerical modeling.. Journal of Geophysical Research - Oceans.

[24] Masseret E, Grzebyk D, Nagai S, Genovesi B, Lasserre B (2009). Unexpected Genetic Diversity Among and Within Populations of the Toxic Dinoflagellate *Alexandrium catenella* Revealed with Nuclear Microsatellite Markers.. Applied and Environmental Microbiology.

[25] Anderson DM, Kulis DM, Doucette GJ, Gallagher JC, Balech E (1994). Biogeography of toxic dinoflagellates in the genus *Alexandrium* from the northeastern United States and Canada.. Marine Biology.

[26] Maranda L, Anderson DM, Shimizu Y (1985). Comparison of toxicity between populations of *Gonyaulax tamarensis* of eastern North American waters.. Estuarine, Coastal and Shelf Science.

[27] Rynearson TA, Armbrust EV (2004). Genetic differentiation among populations of the planktonic marine diatom *Ditylum brightwellii* (Bacillariophyceae).. Journal of Phycology.

[28] Anderson DM, Stock CA, Keafer BA, Bronzino Nelson A, Thompson B (2005). *Alexandrium fundyense* cyst dynamics in the Gulf of Maine.. Deep Sea Research II.

[29] Anderson DM, Kulis DM, Binder BJ (1984). Sexuality and cyst formation in the dinoflagellate *Gonyaulax tamarensis* : Cyst yield in batch cultures.. Journal of Phycology.

[30] Anderson DM, Lindquist NL (1985). Time-course measurements of phosphorus depletion and cyst formation in the dinoflagellate *Gonyaulax tamarensis* Lebour.. Journal of Experimental Marine Biology and Ecology.

