# Supplementary figures and images for: Diversity and Dynamics of a Widespread Bloom of the Toxic Dinoflagellate Alexandrium fundyense
Source: PLoS One. 2011 Jul 29;6(7):e22965. doi: 10.1371/journal.pone.0022965 (PMC3146535; doi:10.1371/journal.pone.0022965)

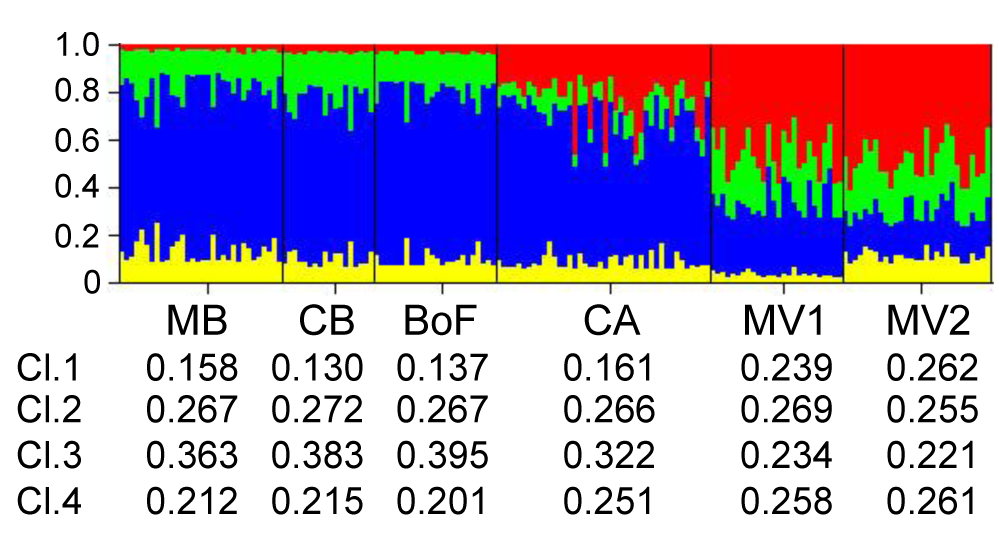

Supplement: Figure S1 — Population structure determined by Bayesian cluster analysis for K = 4. Plots of the statistic ΔK (Evanno et al. 2005) vs. K showed two modes, at K = 2 and K = 4. Results for K = 2 are included in the text, as the presence of two sub-populations is supported by the results of other analyses, whereas the possibility of four sub-populations is inconsistent with the other data. The bar plot shows the proportion of membership in Cluster 1 (red), Cluster 2 (green), Cluster 3 (blue) or Cluster 4 (yellow) for each individual. Samples are delineated by vertical black lines, and the average proportion of membership in each cluster by sample is listed underneath the sample names. (TIF) [file pone.0022965.s001.tif]
