# Supplementary material for: Diversity and Dynamics of a Widespread Bloom of the Toxic Dinoflagellate Alexandrium fundyense
Source: PLoS One. 2011 Jul 29;6(7):e22965. doi: 10.1371/journal.pone.0022965 (PMC3146535; doi:10.1371/journal.pone.0022965)
Supplement: Table S1 — Summary of molecular diversity indices for the six samples analyzed, both per locus and across all loci. The number of unique haplotypes per sample is listed beside the sample name. (DOC) [file pone.0022965.s002.doc]

Supplementary Table 1: Summary of molecular diversity indices for the six samples analyzed, both per locus and across all loci. The number of unique haplotypes per sample is listed beside the sample name.

| **Location** | **Atama15** | **Atama16** | **Atama23** | **Atama27** | **Atama39** | **all loci** |
| --- | --- | --- | --- | --- | --- | --- |
| **CB** | 15 unique haplotypes (83%) | | | | | |
| N | 18 | 18 | 18 | 18 | 18 | 18 |
| N_a_ | 4 | 6 | 4 | 4 | 3 | 4.2 |
| N_e_ | 2.05 | 4.15 | 2.49 | 1.42 | 1.26 | 2.27 |
| R | 10 | 16 | 6 | 6 | 4 | 8.4 |
| H | 0.512 | 0.759 | 0.560 | 0.296 | 0.204 | 0.474 |
| **BoF** | 22 unique haplotypes (92%) | | | | | |
| N | 24 | 24 | 24 | 24 | 24 | 24 |
| N_a_ | 6 | 9 | 3 | 3 | 2 | 4.6 |
| N_e_ | 2.12 | 6.55 | 2.97 | 1.29 | 1.38 | 2.86 |
| R | 14 | 18 | 4 | 4 | 2 | 8.4 |
| H | 0.528 | 0.847 | 0.663 | 0.226 | 0.278 | 0.508 |
| **MB** | 29 unique haplotypes (91%) | | | | | |
| N | 32 | 32 | 32 | 31 | 32 | 31.8 |
| N_a_ | 4 | 8 | 6 | 4 | 3 | 5.0 |
| N_e_ | 1.57 | 5.17 | 4.03 | 1.48 | 1.38 | 2.73 |
| R | 10 | 14 | 18 | 4 | 4 | 10 |
| H | 0.363 | 0.806 | 0.752 | 0.326 | 0.275 | 0.507 |
| **CA** | 35 unique haplotypes (83%) | | | | | |
| N | 42 | 42 | 42 | 42 | 41 | 41.8 |
| N_a_ | 6 | 10 | 6 | 4 | 4 | 6.0 |
| N_e_ | 2.73 | 4.67 | 3.29 | 1.22 | 1.28 | 2.64 |
| R | 26 | 22 | 10 | 6 | 4 | 13.6 |
| H | 0.634 | 0.786 | 0.696 | 0.178 | 0.219 | 0.503 |
| **MV1** | 24 unique haplotypes (92%) | | | | | |
| N | 26 | 26 | 26 | 26 | 26 | 26 |
| N_a_ | 6 | 7 | 6 | 1 | 2 | 5.25 |
| N_e_ | 2.77 | 5.05 | 3.19 | 1.00 | 1.26 | 2.65 |
| R | 7 | 18 | 10 | - | 2 | 9.25 |
| H | 0.639 | 0.802 | 0.686 | 0.000 | 0.204 | 0.466 |
| **MV2** | 24 unique haplotypes (83%) | | | | | |
| N | 29 | 29 | 28 | 27 | 28 | 28.2 |
| N_a_ | 7 | 6 | 6 | 4 | 3 | 5.2 |
| N_e_ | 2.83 | 5.16 | 3.49 | 1.55 | 1.53 | 2.91 |
| R | 28 | 12 | 18 | 4 | 2 | 12.8 |
| H | 0.647 | 0.806 | 0.713 | 0.354 | 0.347 | 0.574 |

N= number of individuals typed at that locus

N_a_ = number of alleles observed per locus

N_e_ = effective number of alleles (Kimura and Crow 1964)

R = allelic range in bp

H = Nei’s (1973) gene diversity
